# The structure of gangliosides hides a code for determining neuronal functions

**DOI:** 10.1002/2211-5463.13197

**Published:** 2021-06-15

**Authors:** Giulia Lunghi, Maria Fazzari, Erika Di Biase, Laura Mauri, Elena Chiricozzi, Sandro Sonnino

**Affiliations:** ^1^ Department of Medical Biotechnology and Translational Medicine University of Milano Italy

**Keywords:** ceramide structure, ganglioside–protein interactions, gangliosides, neuronal functions, oligosaccharide structure, plasma membrane

## Abstract

Gangliosides are particularly abundant in the central nervous system, where they are mainly associated with the synaptic membranes. Their structure underlies a specific role in determining several cell physiological processes of the nervous system. The high number of different gangliosides available in nature suggests that their structure, related to both the hydrophobic and hydrophilic portion of the molecule, defines a code, although not completely understood, that through hydrophobic interactions and hydrogen bonds allows the transduction of signals starting at the plasma membranes. In this short review, we describe some structural aspects responsible for the role played by gangliosides in maintaining and determining neuronal functions.

## Graphical abstract

The biosynthetic pathway of central nervous system sphingolipids: the scheme reports the two main pathways to obtain sphingolipids containing C18‐ or C20‐sphingosine.

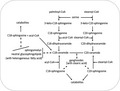

**Abbreviations**
EGFR: epidermal growth factor receptor
GM1: II3Neu5Ac‐Gg4Cer, β‐Gal‐(1‐3)‐β‐GalNAc‐(1‐4)‐[α‐Neu5Ac‐(2‐3)]‐β‐Gal‐(1‐4)‐β‐Glc‐Cer
GM3: II3Neu5AcLacCer, a‐Neu5Ac‐(2‐3)‐b‐Gal‐(1‐4)‐b‐Glc‐(1‐1)‐Cer
NGF: nerve growth factor
Trk: tropomyosin receptor kinase
TrkA: tropomyosin receptor kinase A

Gangliosides are sialic acid‐containing glycosphingolipids that belong to a large group of membrane amphiphilic complex lipids characterized by a wide variety of structures.

They are ubiquitous components of our cell plasma membranes and are particularly abundant and enriched in the brain synapses [1, 2]. On the cell surface, the gangliosides interact with membrane proteins, mostly receptors, thus participating in the modulation of different cellular functions, including neuronal development and maturation, synaptic activity, and ion balance [3]. Different cellular processes require specific ganglioside structures that are the synergic result of the expression and activity of both anabolic and catabolic enzymes [4].

The research on gangliosides started at the end of 19th century by J.L.W. Thudichum [5], who extracted new unknown compounds from the ganglion of human brains, which he named ‘Gangliosides’. For many years, the structure and the role of gangliosides remained obscure, associating them to the enigma of sphinx. Progress in this field was extremely slow, and the correct structure of sphingosine, the central portion of the ganglioside lipid moiety, whose name root still recalls the ‘sphinx,’ was established only after half a century, in 1947 [6]. However, it was necessary to wait until 1963 to know the first total structure of a complex ganglioside, such as GM1 [II3Neu5Ac‐Gg4Cer, β‐Gal‐(1‐3)‐β‐GalNAc‐(1‐4)‐[α‐Neu5Ac‐(2‐3)]‐β‐Gal‐(1‐4)‐β‐Glc‐Cer] [7].

Subsequently, the advancement of analytical chemistry and the availability of high‐resolution physical‐chemical procedures allowed to identify hundreds of different structures of neutral glycosphingolipids and gangliosides [4]. Because, as mentioned earlier, the structure of a ganglioside is associated with its biological role, the current focus is to dissect the bioactive role that links a specific ganglioside structure to the modulation of intracellular events.

## Ganglioside structures

**Fig. 1:**
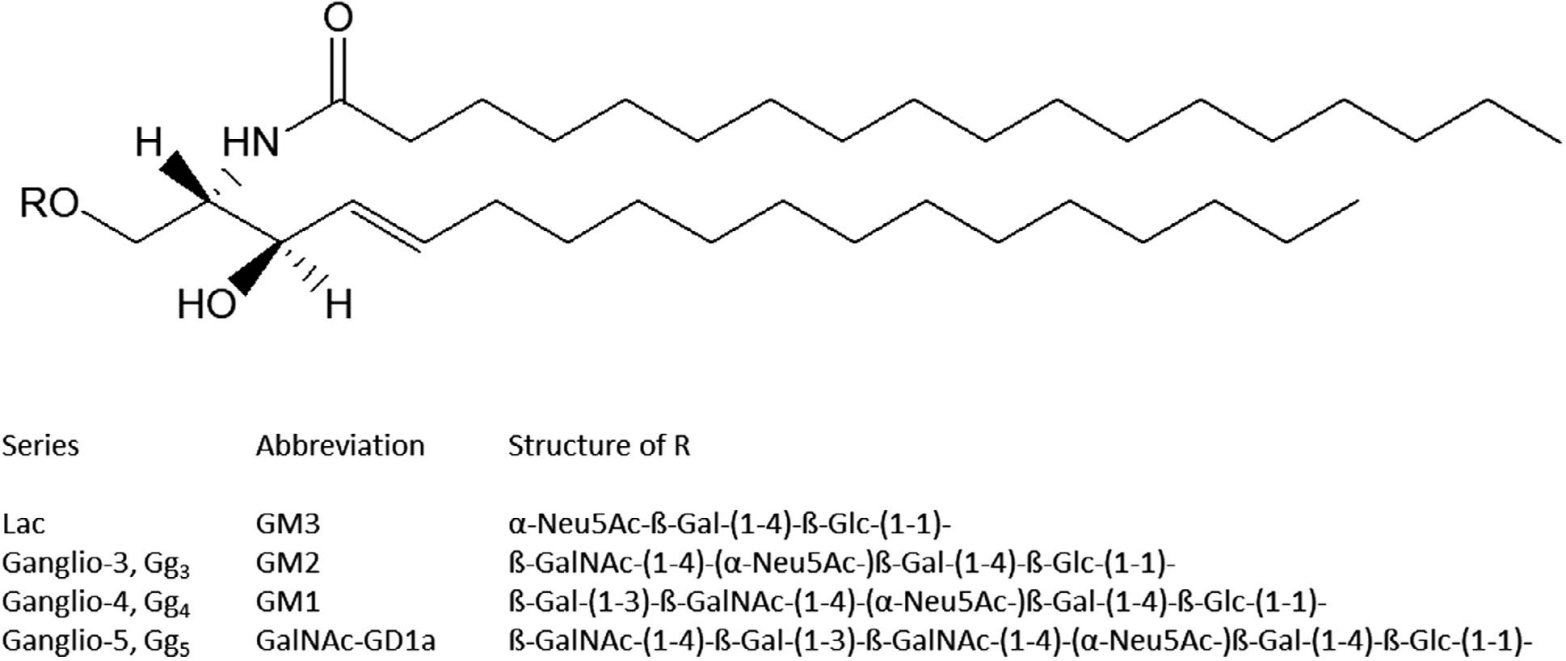
Structure of ganglioside ceramide containing C18‐sphingosine and stearic acid. The figure reports some R groups linked to the primary ceramide hydroxyl group.

Gangliosides are amphiphilic molecules that contain lipid and carbohydrate moieties. The lipid moiety, named ceramide, contains two long‐alkyl chains: a long‐alkyl chain amino alcohol, mainly the (2*S*,3*R,4E)‐*2‐amino‐1,3‐dihydroxy‐octadec/eicos‐4‐ene, whose trivial name is sphingosine [8], connected to a fatty acid by an amide linkage [9, 10]. In the nervous tissue, where gangliosides are 10 times more abundant than in any other tissue, the stearic acid represents more than 90% of the total fatty acid content [10, 11] (Fig. 1).

**Fig. 2:**
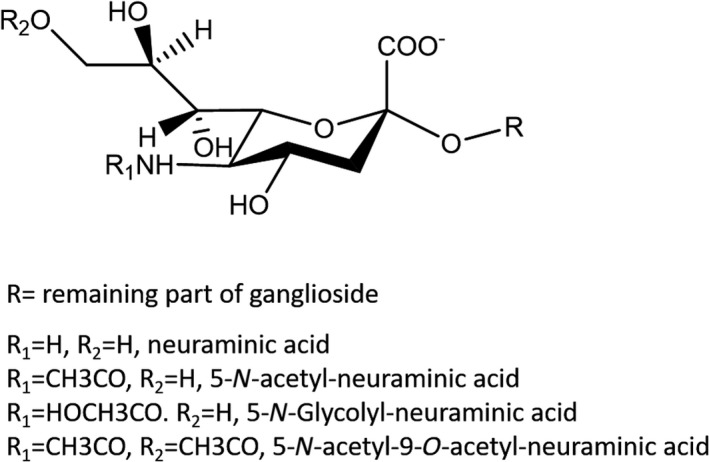
Structure of sialic acid. The possibilities for R, R1 and R2 are reported.

The hydrophilic head group of the main human neuronal gangliosides contains from one to five neutral carbohydrates, displaying β‐configuration, linked to each other by glycosidic linkages. One to six sialic acids, in α‐configuration, are linked to the neutral oligosaccharide. The trivial name ‘sialic acid’ identifies all the derivatives of neuraminic acid, whose chemical name is 5‐amino‐3,5‐dideoxy‐d‐*glycero*‐d‐*galacto*‐non‐2‐ulopyranosonic acid [12, 13]. The three most represented structures of sialic acid are 5‐*N*‐acetyl‐, 5‐*N*‐acetyl‐9‐*O*‐acetyl and 5‐*N*‐glycolyl‐neuraminic acid, but the latter is absent in normal human tissues [14, 15] (Fig. 2). In human brain gangliosides, about 10% of the associated sialic acid is the 9‐*O*‐acetyl‐*N*‐acetylneuraminic acid [16, 17]. Ester linkages in polysialyl chain‐containing compounds also have been characterized [18, 19].

## Gangliosides and lipid rafts

The structure of both hydrophobic and hydrophilic moieties of gangliosides displays several features that drive the formation of membrane domains, today worldwide called ‘lipid rafts’ (see reviews [3, 20, 21, 22, 23, 24, 25, 26] for detailed information on gangliosides and lipid rafts).

The terminology ‘lipid rafts’ identifies a group of different membrane domains highly enriched in sphingolipids, cholesterol and dipalmitoylphosphatidylcholine, but containing a smaller amount of proteins [27]. Thanks to their structural features, gangliosides provide the necessary forces for the organization of physiological lipid rafts with different characteristics. Indeed, the big hydrophilic oligosaccharide, together with the negatively charged sialic acid and the multiple conformations of glycosidic linkages, implies that a ganglioside monomer occupies a very large area on the cell surface. This feature thermodynamically favors the ganglioside aggregation in the fluid membrane surface. In addition, gangliosides can interact each other through side‐by‐side hydrogen bonds mediated by ˜40–50 water molecules that act as linking bridges between the chains [28].

Gangliosides remain stably inserted into the outer layer of plasma membranes through lipid–lipid interactions. The position of gangliosides at the water–lipid interface is stabilized by hydrogen bonds with neighboring glycerophospholipids, particularly with the more rigid ones containing the short and saturated palmitic acid, involving both the amide proton and the carbonyl of ceramide [3].

These interactions make the ganglioside‐enriched membrane less fluid and inclined to incorporate cholesterol. The following sphingolipid–cholesterol hydrophobic interactions segregate the hydrophobic and rigid portion of membrane proteins, forming a stable membrane raft considered functional for several physiological cell processes.

## The code hidden in the ganglioside ceramide structure

The ceramide of gangliosides has often been considered as a barely structural anchor necessary for maintaining the ganglioside inserted into the plasma membrane outer layer. In contrast, the biological implications of ceramide released by sphingomyelin catabolism at the cell surface has attracted a lot of interest for its capability to induce cell apoptosis [29, 30]. However, this has been later verified to be valid also for the ceramide produced by gangliosides by the sequential activity of plasma membrane‐associated glycohydrolases [31]. As for sphingomyelinase, glycohydrolases are activated by high‐energy radiations and by the activity of plasma membrane hydrogen pumps [32, 33].

The peculiarity of neuronal ganglioside ceramide resides in its structure. The fatty acid linked to the sphingosine of this ceramide is predominantly stearic acid, representing 90–95% of the total fatty acids, together with a scant quantity of palmitic and the 20‐carbon arachidic acid [10]. The 24‐carbon lignoceric acid, present in the extra‐nervous system gangliosides, neutral glycosphingolipids and sphingomyelin, is difficult to detect. The same is true for unsaturated fatty acid.

A second characteristic of the neuronal ganglioside ceramide is the structure of sphingosine. Sphingosine is the general trivial name of a basic long‐alkyl chain. Among the sphingolipids, sphingomyelin, neutral glycosphingolipids and gangliosides, the most represented sphingosine molecule is the (2*S*,3*R,4E)‐*2‐amino‐1,3‐dihydroxy‐octadec‐4‐ene currently named and known as C18‐sphingosine [6]. Many other structures have been reported to exist in nature, differing in length of the alkyl chain, degree of unsaturation, number of hydroxyl groups and presence of lateral methyl groups [34]. In general, these structures are present in very minor amounts in mammalian gangliosides, except for the (2*S*,3*R,4E)‐*2‐amino‐1,3‐dihydroxy‐eicos‐4‐ene, the C20‐sphingosine. C20‐sphingosine is characteristic of neuronal gangliosides and can be particularly abundant [9].

**Fig. 3:**
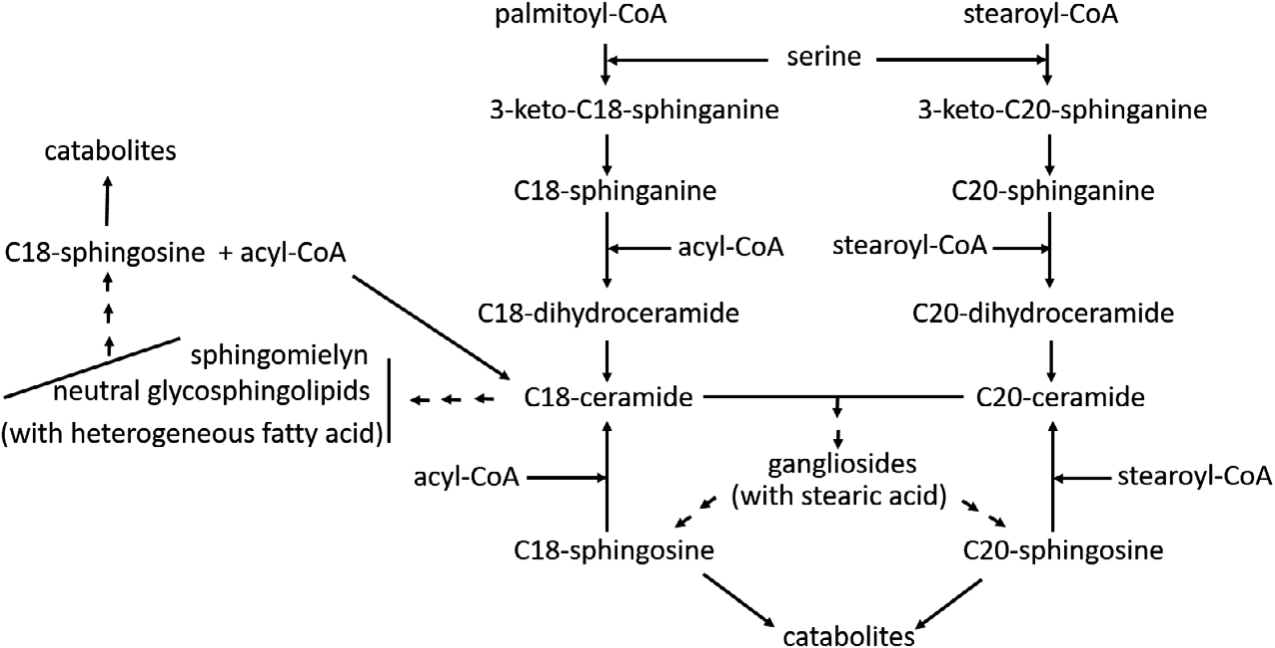
Scheme of the biosynthetic process for C18‐ and C20‐sphingosine containing sphingolipids. Neuronal gangliosides contain both C18‐ and C20‐sphingosine, whereas neutral glycosphingolipids and sphingomyelin contain the sole C18‐sphingosine. Neuronal gangliosides contain quite exclusively stearic acid, whereas neutral glycosphingolipids and sphingomyelin are heterogeneous in the acyl chain.

The key enzyme for the biosynthesis of ceramide containing C18‐ or C20‐sphingosine is the acyl‐CoA‐serine acyltransferase, or 3‐ketosphinganine synthase (Fig. 3). The biosynthesis of ceramide proceeds in the endoplasmic reticulum through the pathway acyl‐CoA + serine → 3‐keto‐sphinganine → sphinganine → dihydroceramide → ceramide. Ceramide is then transferred to the Golgi apparatus, where the glycosyltransferases proceed with the synthesis of the glycosphingolipid oligosaccharide chain and the sphingomyelin synthase with the synthesis of sphingomyelin. According to the earlier pathway, sphinganine, that is, the saturated long‐chain base, is synthesized instead of sphingosine. The double bond is introduced after the addition of the fatty acid to sphinganine. The cell sphingosine is a catabolic product highly recycled for the biosynthesis of sphingolipids, and it leads in part to total catabolism [4].

The 3‐ketosphinganine synthase uses palmitoyl‐CoA to yield C18‐sphinganine and stearoyl‐CoA to yield the C20‐sphinganine. Both activated fatty acids are available in large quantities at any stage of the nervous system, but in cells the ratio between ganglioside species containing C18‐sphingosine and those containing C20‐sphingosine varies with the differentiating and aging processes [35]. At fetal stage and at birth, the C20 ganglioside species are absent or very scant. After that, they progressively increase to become more abundant species in adult and aged mammals [36, 37].

Data on the expression of 3‐ketosphinganine synthase along the span life are not available, but the activity of the enzyme on palmitoyl‐CoA and stearoyl‐CoA in cultured cells is correlated with the C18‐ and C20‐ganglioside species content [35]. The enzyme has practically no activity on stearoyl‐CoA at the beginning of differentiation. Then, this activity appears and progressively increases, with a parallel decrease of the enzyme activity on palmitoyl‐CoA [35, 38].

The length of the ganglioside sphingosine is of great importance in defining the length and hydrophobic volume of the ceramide moiety [39]. These two parameters modulate the geometrical and amphiphilic properties of the ganglioside molecule (i.e. its packing parameter) [39], varying the fluidity, the thickness and the organization of the membrane domain in which gangliosides are inserted together with specific proteins. Such a control is very important in modulating and addressing the lipid–protein interactions [40]. In addition to this, we recall that the length of the sphingosine determines the fluctuation of the total ganglioside molecule at the water–lipid interface and extension of the head group over the surface. Thus, it is possible to speculate on the involvement of single‐ganglioside species containing C18‐ or C20‐sphingosine in the ability to modify membrane features, thus determining changes in ganglioside–protein interactions and in physiological processes.

## The code hidden in the ganglioside oligosaccharide structure

The oligosaccharide moiety of gangliosides protrudes into the cellular aqueous environment by 20–25 Å [39]. This extension is much lower than that of the extracellular portion of proteins. The sphingolipid‐enriched lipid rafts contain a small number of proteins, thus allowing side‐by‐side interactions between the oligosaccharide structures [3]. Some water molecules, at the water–lipid interface, surround the oligosaccharide groups interacting with the hydroxyl groups of single‐carbohydrate units. This is peculiar for carbohydrate–carbohydrate or carbohydrate–protein interactions mediated by water bridges [41]. In addition to this, we recall the role played by sialic acid in the ganglioside–protein interaction processes. The sialic acid is negatively charged, because of its pK_a_ value near 2, thus attracting positive groups, ions and water as well, and allowing strong ionic interactions with the protein amino acids. From a chemical point of view, considering together the number of sugars per chain, their repeatability, the possible branching chains, the sugar configuration and the linkage position, the number of different ganglioside oligosaccharides could be incredibly wide [42]. However, the evolution has determined for the human brain a specific biosynthetic pathway that leads to nearly 20 ganglioside species [43]. Of course, considering that each species is heterogeneous in its ceramide moiety, the number of brain types is higher. This allows to arrange a high number of molecules that like different keys can open a series of locks; that is, different ganglioside–protein interactions regulate diverse neuronal processes.

Information about the ganglioside–protein interactions and the capability of gangliosides to modulate the activities of proteins is very wide, but only a few specific interactions between gangliosides and receptor proteins have been studied in detail. This concerns mainly ganglioside GM3 [II3Neu5AcLacCer, a‐Neu5Ac‐(2‐3)‐b‐Gal‐(1‐4)‐b‐Glc‐(1‐1)‐Cer] and GM1. GM3 interacts with the epidermal growth factor receptor (EGFR) and with the insulin receptor. In both cases, the interaction negatively modulates the receptors, leading to cell growth inhibition in one case and insulin resistance in the other one [44, 45, 46, 47, 48, 49, 50]. Overall, GM3 exerts its biological activity in a nonneuronal environment.

**Fig. 4:**
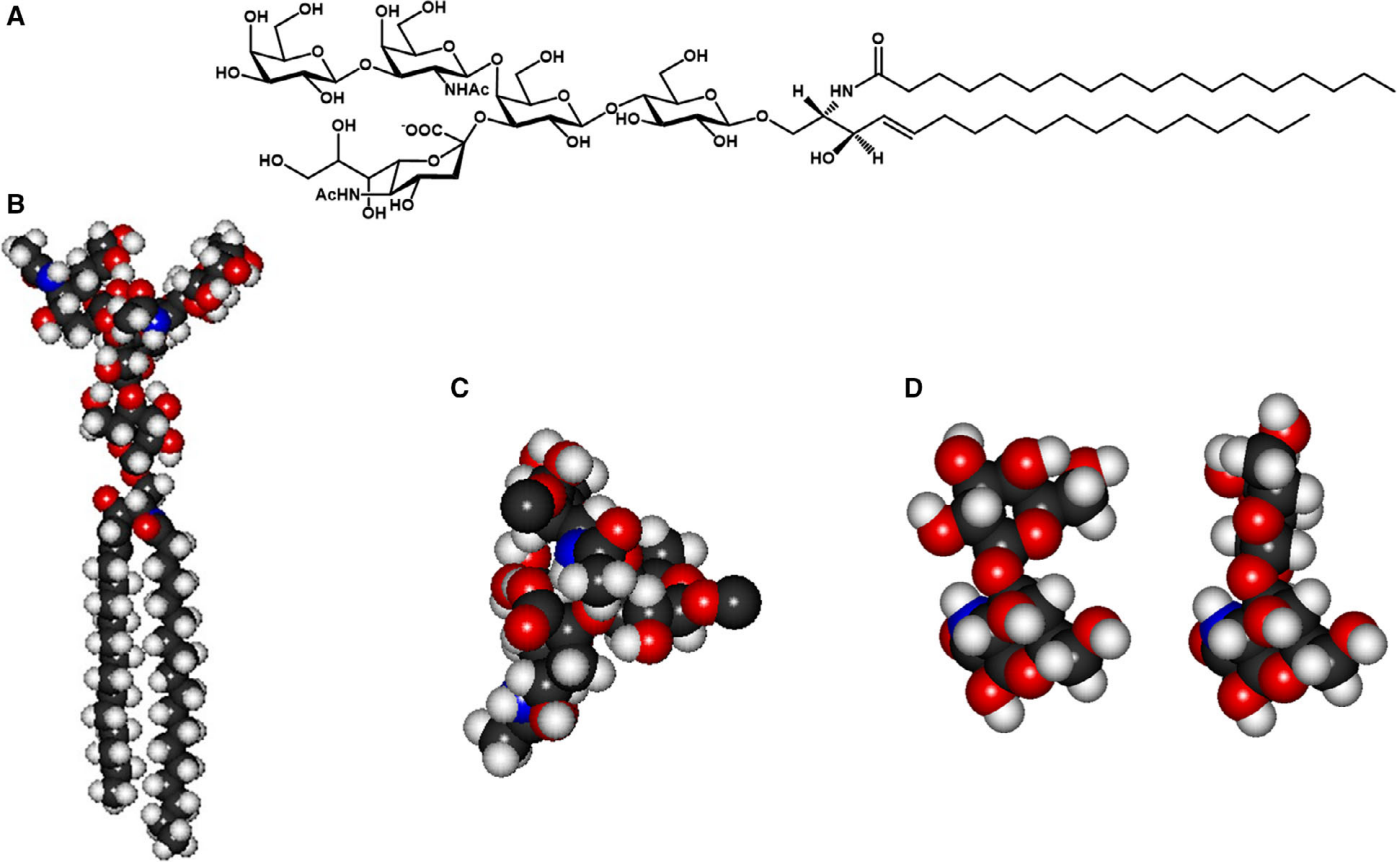
GM1 ganglioside. Representation of the GM1 structure (A). Minimum energy conformation structure of GM1 (B). Conformation of the GM1 trisaccharide‐β‐GalNAc‐(1‐4)‐[α‐Neu5Ac‐(2‐3)‐]β‐Gal‐; the GM1 trisaccharide behaves as single rigid block because of interactions between the lateral chain and carboxyl group of sialic acid with *N*‐acetylgalactosamine (C). The two main conformations of the GM1 external disaccharide β‐Gal‐(1‐3)‐β‐GalNAc‐ (D).

On the other side, GM1 (see Fig. 4 for GM1 structure) strongly influences specific neuronal functions by interacting with the tropomyosin receptor kinase (Trk) A (TrkA) receptor [51, 52, 53] and with the intramembrane GFRα1 protein connected with the RET receptor [54, 55].

The interaction between GM1 and TrkA has been a matter of interest among scientists for many years. There is currently solid evidence demonstrating the specific involvement of GM1 in making the conformation of TrkA suitable for interacting with the nerve growth factor (NGF) ligand.

GM1 binds Trk family receptors with high affinity in cultured cells and stimulates Trk receptor kinase activity, resulting in receptor autophosphorylation associated with the activation of the downstream signal transduction cascade that results in various cellular responses, such as modulation of mitochondrial activity and availability of cytosolic calcium [56, 57]. Recently, it has been suggested that TrkA and GM1 are not components of the same membrane domain, but that the TrkA can approach the ganglioside oligosaccharide forming the trimeric complex TrkA–NGF–GM1 [58]. The GM1–TrkA interaction does not involve the GM1 ceramide through hydrophobic interactions. Only the GM1 oligosaccharide is involved in the interaction with TrkA, as demonstrated with a series of photoactivable and tritium‐labeled GM1 derivatives, probably suggesting the reason why it is not necessary for the two actors to be in the same domain: proximity is enough [58].

The GM1–Trk interaction is probably obtained or favored by the action of the plasma membrane‐associated sialidase Neu3 that can change the membrane ganglioside composition, locally modifying the GM1 content [59]. This is also confirmed by the expression and activity of Neu3 on the neurite formation [53].

The interaction between GM1 and TrkA is highly specific, and the removal of the sialic acid or of the external galactose abolishes any possibility of interaction and activation of the TrkA receptor [48].

**Fig. 5:**
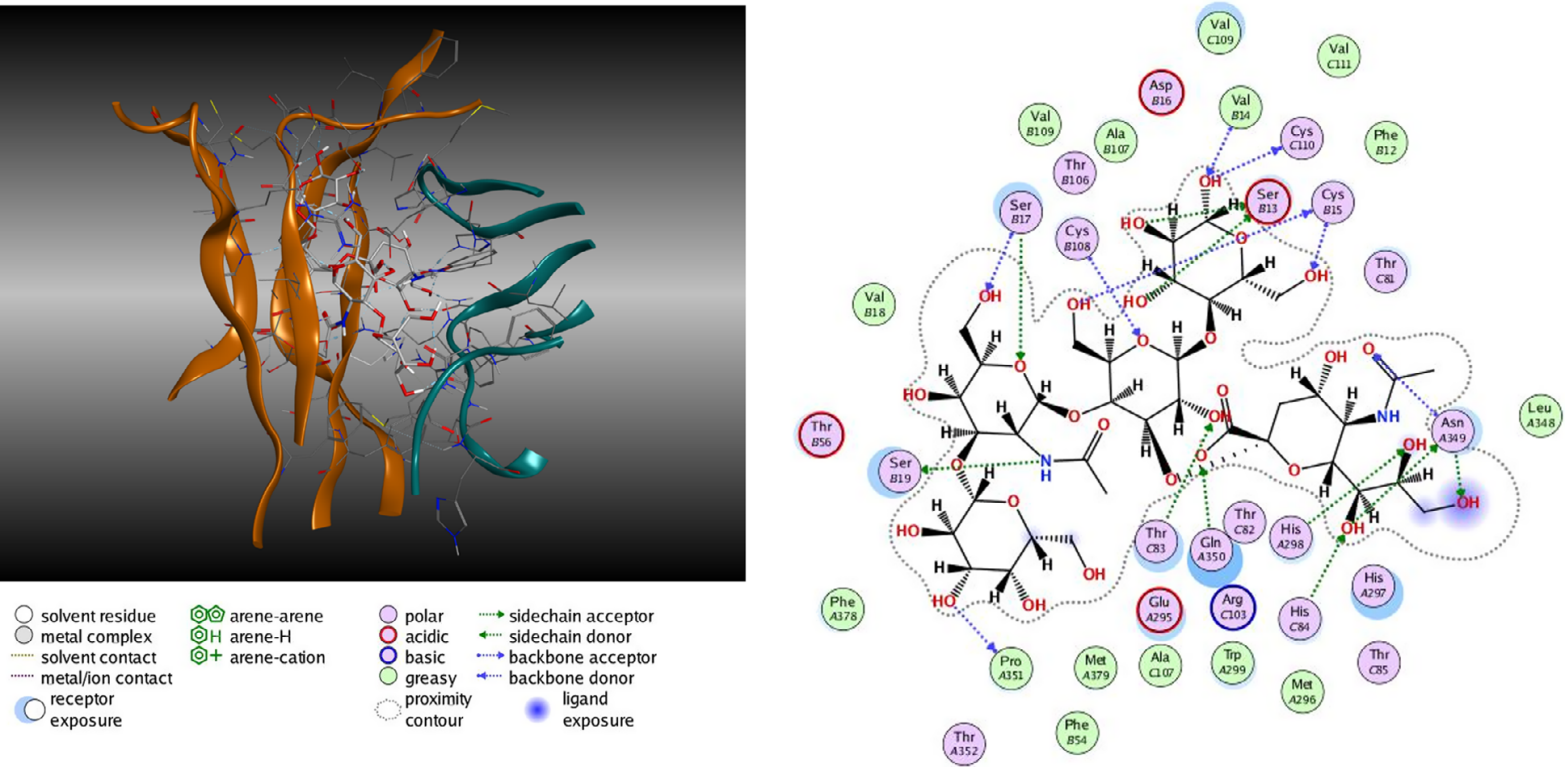
Representation of TrkA–GM1 oligosaccharide complex and prediction of point of interaction among the TrkA amino acids and the GM1 oligosaccharide sugars.

By molecular modeling it results that GM1 favors the NGF–TrkA interaction and dimerization by increasing the complex stability. The crystallized structure of the NGF–TrkA complex is characterized by a pocket in which the GM1 oligosaccharide perfectly fits. By docking analysis performed using the sole GM1 oligosaccharide, a series of possible interactions between the oligosaccharide and protein amino acid groups has been found (Fig. 5). Following this, the energy associated to the TrkA–GM1 complex declines from −6.6 to −11.5 kcal·mol^−1^ when NFG belongs to the complex [60].

In conclusion, TrkA requires both GM1 and NGF to be activated, inducing the phosphorylation cascade responsible for several neuronal processes. NGF is released by the cells that control its availability for the interaction with TrkA, and GM1 is an endogenous component of our membranes whose absence does not allow proper TrkA functioning [61], even in the presence of NGF, thus preventing a healthy life. According to this, the true NGF receptor seems to be the TrkA–GM1 complex. The release of the soluble NGF from the cells results in the formation of a big trimeric NGF–TrkA–GM1 complex allowing TrkA dimerization, followed by the autophosphorylation of the receptor cytosolic portion.

## Conflict of interest

The authors declare no conflict of interest.

## Author contributions

SS: conceptualization and first draft of the paper. LM and EC: preparation of figures. GL, MF and EDB: contribution to the writing and final revision.
